# Natural Conditions Override Differences in Emergence Rhythm among Closely Related Drosophilids

**DOI:** 10.1371/journal.pone.0083048

**Published:** 2013-12-11

**Authors:** Priya M. Prabhakaran, Joydeep De, Vasu Sheeba

**Affiliations:** Behavioural Neurogenetics Laboratory, Evolutionary and Organismal Biology Unit, Jawaharlal Nehru Centre for Advanced Scientific Research, Bangalore, Karnataka, India; University of Ferrara, Italy

## Abstract

Previous studies on adult emergence rhythm of *Drosophila melanogaster* (DM) done under semi-natural conditions have shown that emergence is correlated to daily changes in temperature, humidity and light at dawn. Recently we showed that under laboratory conditions *D. ananassae* (DA), a closely related species of DM exhibits patterns in its activity/rest rhythm distinct from the latter. Here, we report the results of a study aimed at examining whether this difference in activity/rest rhythm among species extends to other circadian behaviours such as the adult emergence rhythm under a more natural environment with multiple cyclic time cues. We monitored the adult emergence rhythm of recently wild-caught DM and DA populations in parallel with those of a related species *D. malerkotliana* (DK), both in the laboratory and under semi-natural conditions. We find that although DM, DK and DA showed marked difference from one another under laboratory conditions, such differences were not detectable in the emergence behaviour of these three species under semi-natural conditions, and that they respond very similarly to seasonal changes in the environment. The results suggest that seasonal changes in temperature and humidity contribute largely to the variation in adult emergence waveform in terms of gate width, phase and amplitude of the peak and day-to-day variance in the timing of the emergence peak. In all three species, seasons with cooler and wetter conditions make the rhythm less tightly gated, with low amplitude peak and high day-to-day variation in timing of the peak of emergence. We show that in nature the emergence rhythm of DM, DK and DA is strongly influenced by environmental factors such that in a given season all of them exhibit similar time course and waveform and that with the changing season, they all modify their emergence patterns in a similar manner.

## Introduction

 In most organisms daily environmental cycles modulate the rhythmic behaviours controlled by circadian clocks thus enabling them to maximally exploit resources and to minimize the effects of adverse conditions [1,2]. We have shown previously that sympatric species *Drosophila melanogaster* (DM) and *D. ananassae* (DA) differ in several features of their activity-rest rhythm [3]. DM exhibits a bimodal activity pattern whereas activity of DA is skewed towards morning, which persists under a range of photoperiods in the laboratory. Under laboratory conditions, DA is most active at the beginning of the light phase after which its activity tapers off as the day progresses. Thus, unlike DM, DA does not exhibit ‘siesta’ during midday [3]. Such differences also persisted across a range of seasons when assayed under semi-natural conditions [4]. We hypothesized that these two relatively recently diverged sympatric species of *Drosophila* occupy different temporal niches due to the differences in their underlying circadian clocks. In the present study, we aimed at examining whether the above mentioned differences in activity/rest rhythm extends to another circadian behaviour - adult emergence rhythm. Although it has long been hypothesised that emergence of fruit flies peaks at dawn to coincide with maximum humidity levels [5], there is no clear evidence for such an adaptive response. Furthermore, it is known that there are other insects whose emergence is restricted to daytime when humidity levels are low [2]. Hence we examined the pattern of adult emergence of DA whose activity is phased predominantly towards the early part of the day, a time during which DM activity falls dramatically. These studies were carried out along with another sympatric species *D. malerkotliana* (DK) under laboratory conditions (LAB) and under semi-natural conditions (SN) that were created in an outdoor facility [6]. DK flies exhibited an activity /rest rhythm very similar to DM flies under LAB LD and DD regimes (Figure S1). For each species large random mating populations that have been relatively recently caught from localities in Bangalore (12°59'N 77°35'E) and maintained in the LAB were used.

We also attempted to explore how environmental factors across seasons shape adult emergence of Drosophilids by conducting studies at five different times of the year. Although at this location we do not experience large changes in photoperiod, seasons are marked by changes in absolute values of temperature and relative humidity as well as in the day/night variation of these environmental variables. We refer to the studies done outside the laboratory as semi-natural conditions throughout, since we acknowledge that our method does not capture the behaviour under truly natural conditions. In DM, the act of adult emergence has been shown to be clock-controlled and entrainable to daily cycles of light and temperature [2]. Under standard LAB protocols (12:12 h light/dark cycles, henceforth, LD), emergence is gated in such a manner that it is largely restricted to daytime with a sharp peak around dawn [7]. One popular hypothesis regarding the circadian regulation of emergence at dawn stresses upon the importance of temperature and humidity as key factors [1]. Recently we have demonstrated that under SN most of the emergence occurs during early morning which is also the time when temperature is low and relative humidity is high [6]. Similar to what has been seen with activity-rest rhythm [4,8-11], several features of emergence rhythm also differ between SN and LAB [6]. Under SN, the gate-width of adult emergence rhythm and nighttime emergence of flies decreased significantly compared to LAB, which suggests that natural environmental cycles probably exert greater pressure upon the gating of this rhythm than those of the LAB. This may be due to the presence of multiple, gradually varying time-cues (Zeitgebers) of relatively higher amplitude in SN unlike LAB, where the only Zeitgeber is white light (ON/OFF) of relatively low intensity (~100 lux), whose wavelength composition is also constant.

A preliminary attempt to examine the effect of seasonal variations in environmental factors on adult emergence of CS strain of DM flies revealed that during harsh conditions, much of the emergence occurs between late night to early morning, whereas during milder weather conditions, emergence continues until afternoon [6]. The study also found that under milder conditions, the number of CS flies emerging during the day was correlated to daily changes in the light intensity but not with temperature or humidity [6]. During harsh conditions, the same was correlated to daily changes in humidity and temperature but not to light [6]. A careful inspection of emergence during dawn revealed that the number of flies emerging during the morning hours (between 4-10 h) is positively correlated with changes in the average light intensity [6]. Since these correlations were based on only one strain of DM (*w* ^*1118*^) it has only a limited value in revealing how environmental variables influence the emergence rhythm of flies. Therefore, to obtain greater insight on how natural environment influences emergence, we used a comparative approach and examined this rhythm in three Drosophilid species under SN. The assays were spread across six months with greater variation in environmental factors than before [6], including the coolest and warmest times of the year in Bangalore, India.

Previous studies have shown that differences among strains of DM in activity/rest and emergence rhythm were significantly reduced when studied under SN compared to LAB [6,9]. Flies carrying a mutation in the *period* gene (*per* ^*0*^), an important circadian clock gene showed rhythmic emergence in SN, much like wild-type strain [6], and their activity/rest pattern was also very similar - especially with respect to the morning component [9]. However, DM flies that have evolved precise circadian clocks [12] showed greater divergence in emergence pattern from their controls when assayed under SN. A similar enhancement of difference in phasing of the peak of emergence was seen under SN between two sets of populations of flies selected to emerge either in the morning or late in the evening when studied under SN [13]. We asked whether there is any difference in the emergence rhythm among closely related species of *Drosophila* in the LAB, if yes, whether that extends to different seasons in SN as well. We found that even though the three species (DM, DK and DA) showed differences in their emergence rhythm in the LAB, under SN such differences were considerably reduced. These results suggest that factors in the natural environment that influence emergence have an overriding effect on this behaviour which nullifies any functional difference in rhythmic behaviour that each species is able to exhibit in the LAB.

## Materials and Methods

### Fly strains

DM, DK and DA flies were caught from wild using fruit-traps as bait and net sweeps within Bangalore, India, between 2004-2005. To prevent random genetic drift and founder effects, these flies were maintained as large random mating populations with roughly 1:1 sex ratio of ~1200 individuals. Stocks were maintained under LD12:12 (~1.5 W/m^2^) conditions at constant temperature (~25 °C) and humidity (~70%) with a discrete-generation cycle of 21 days on cornmeal medium.

### Adult emergence assay

Assays were conducted in three different conditions- laboratory Light 12h: Dark 12h (LD) at 25 °C, laboratory constant darkness (DD) at 25 °C and semi-natural (SN) conditions. From population cages of DM, DK and DA, approximately 300 eggs were collected and placed into each glass vial with ~10 ml of food. Ten such vials were used per species per condition. Vials were monitored for darkening of pupae and emergence of the first fly at approximately 6 h intervals. Upon emergence of the first few flies, the vials were monitored at 2 h intervals and adults were cleared from the vials and counted. Assays under SN were conducted in an outdoor enclosure kept under a canopy within JNCASR campus [6] during five different months- March, April, November and December-2012 and February-2013. In parallel the daily profiles of light, temperature, and humidity under SN were monitored using DEnM, Trikinetics, USA. Unlike light intensity and temperature, humidity profile outside the vials is likely to be different from what the developing flies experience inside the vials, which were also plugged with cotton.

### Analysis of emergence data

Emergence profiles of each species were plotted by averaging daily profiles of 10 replicate vials for successive days. To compare emergence rhythm across species in laboratory conditions, we quantified several properties of the rhythm - gate-width, onset of emergence, peak phase, variance in peak timing and percentage of nighttime emergence (LD) for each vial. Gate-width was estimated as the time-interval between start and end of emergence in one complete cycle (using 5% of total emergence in that cycle as cut-off). The onset of emergence was determined from the daily profiles of each vial as the first bin above the 5% cut-off. Peak(s) of emergence were determined from daily profiles of each vial using analysis of variance (ANOVA) with time-point as fixed factor, followed by post-hoc multiple comparisons using Tukey’s HSD test. Variance in peak-timing was estimated as day-to-day variation in timing of emergence-peak in each vial, averaged over replicate vials. One-way ANOVA followed by post-hoc multiple comparisons using Tukey’s HSD test was performed to evaluate statistically significant differences across species in LD and DD separately for gate width, onset of emergence, period, peak phase, peak amplitude and nighttime emergence. Under SN conditions, to compare emergence rhythm across species and months, we quantified several parameters of the rhythm - gate-width, phase of onset of emergence, peak timing, % nighttime emergence and variance in peak timing. The duration from 22:00 h to 4:00 h was considered as ‘nighttime’ since the DEnM monitor did not register values above 0 lux light intensity. Percentage nighttime emergence was averaged across vials and cycles. The gate-width, phase of onset of emergence, peak phase, day-to-day variance in peak phase, peak amplitude and nighttime emergence data were subjected to separate two-way ANOVA to examine the main effect and interaction of species and assay month. Non-parametric Spearman’s rank order correlation test was applied on the following pairs of datasets : gate width versus maximum, minimum, average day and average night temperature and humidity (T_max_, T_min,_ T_ave day,_ T_ave night,_ H_max_, H_min,_ H_ave day_ and H_ave night_); gate width versus maximum and average day light intensity (L_max_ and L_ave day_); onset phase of emergence versus temperature, humidity and light values (T_max_, T_min,_ T_ave day,_ T_ave night,_ H_max_, H_min,_ H_ave day_, H_ave night,_ L_max_ and L_ave day_); peak phase of emergence versus temperature, humidity and light values (T_max_, T_min,_ T_ave day ,_ T_ave night,_ H_max_, H_min,_ H_ave day_ , H_ave night,_ L_max_ and L_ave day_). Error bars shown in the emergence profiles are ± SEM. Error bars in all other graphs are 95% Confidence Interval (± 95% CI). All statistical tests were done using STATISTICA-7 (StatSoft Inc., USA) with level of significance set to *p* < 0.05.

## Results

### Under laboratory conditions adult emergence rhythm of the three Drosophilidc species show differences in temporal distribution

**Figure 1:**
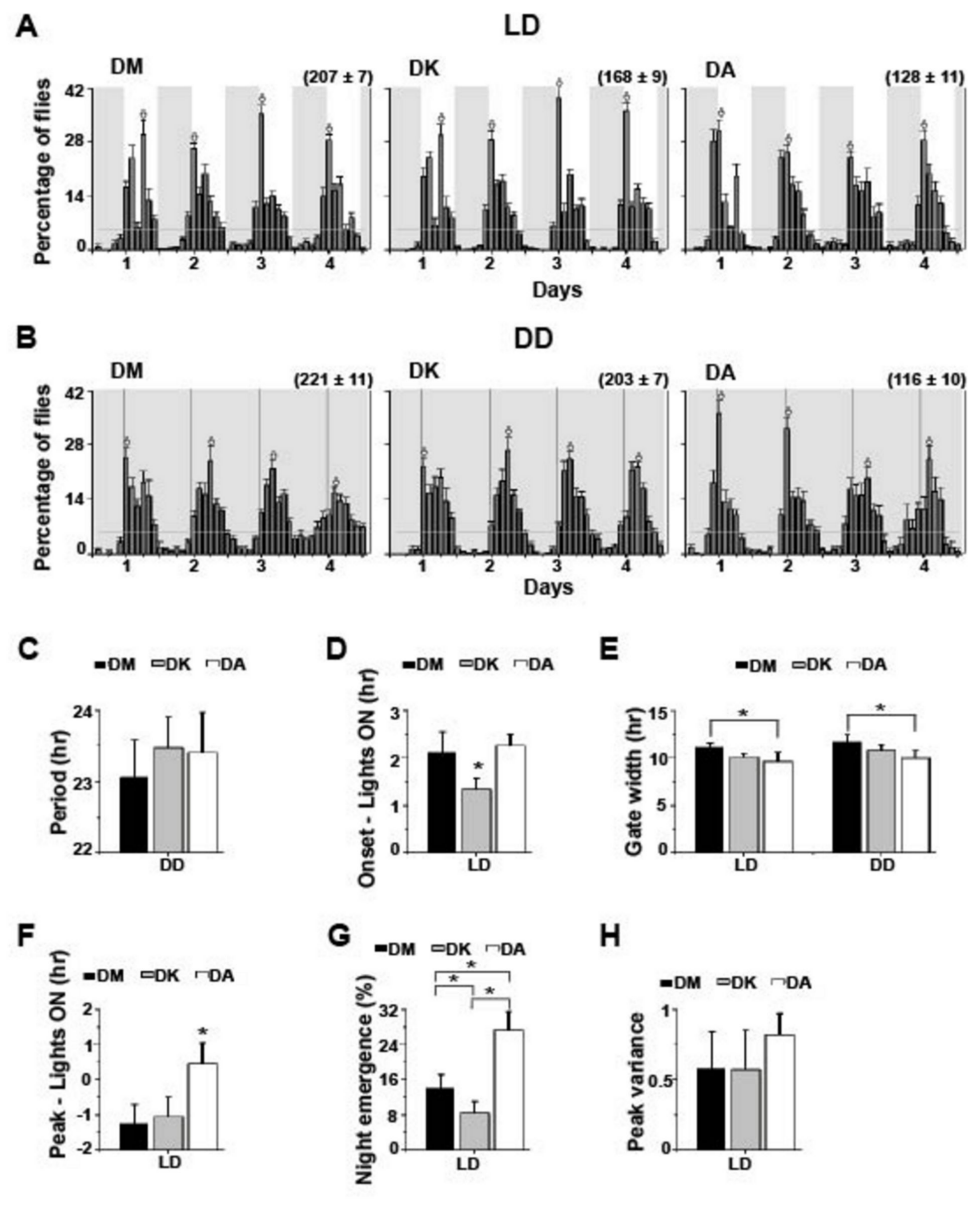
Adult emergence rhythm of *D. melanogaster* differed from *D. ananassae* under laboratory conditions. (A) Average adult emergence profiles (percentage of flies emerged/ 2 h ± SEM, for each species averaged across 10 vials) of *D. melanogaster* (DM), *D. malerkotliana* (DK) and *D. ananassae* (DA) under LD 12:12. Grey shaded areas in the average profiles indicate darkness and 5% of the emergence is denoted by the grey horizontal line. Arrows indicate the peak for each cycle in this average profile. Values in parentheses indicate the total number of flies emerged averaged across 10 vials (± SEM). (B) Average adult emergence profiles of DM, DK and DA under DD (averaged across 10 vials ± SEM). Dotted lines indicate phase of lights-ON in the previously experienced LD regime. All other details are same as in panel A. (C) Average period based on onset of emergence (± 95% CI, averaged across vial) of DM, DK and DA under DD. (D) Average phase of onset of emergence (Time of onset - lights-ON ± 95% CI, averaged across 10 vials) of DM, DK and DA under LD. (E) Average gate-width of emergence (± 95% CI, averaged across 10 vials) of DM, DK and DA under LD and DD. (F) Average phase of the peak of emergence (Time of peak - lights-ON ± 95% CI, averaged across 10 vials) of DM, DK and DA under LD. (G) Average percentage of nighttime emergence (± 95% CI, averaged across 10 vials) of DM, DK and DA under LD. (H) Average day-to-day variation in peak emergence under LD estimated for each vial (*n* = 10 vials). *p < 0.05.

This being the first report of emergence rhythm for the two species DA and DK, we began by examining whether this behaviour is indeed rhythmic and whether the rhythm is similar to the well-studied species DM. All three species DM, DK and DA showed robust entrainment of emergence rhythm to LD cycles with period indistinguishably close to 24 h (DM- 23.8 ± 0.2 h, DK- 23.7 ± 0.1 h, DA- 24.2 ± 0.2 h; Figure 1A), and the rhythms persisted under DD with no species-specific difference in free-running period (Figure 1B, C). Under LD, the onset of emergence was significantly delayed for DK compared to DM and DA (*F*_2,27_ = 11.3, *p* < 0.0003; Figure 1A, D) as determined from vial-wise data (see methods). Both under LD and DD conditions DA exhibited a significantly narrower gate-width of adult emergence than DM (LD-F_2, 27_ = 7.1, *p* < 0.003; DD-F_2,27_ = 7.2, *p* < 0.003; Figure 1E), while this difference from DK was statistically not significant. DA also exhibited advanced peak of emergence compared to DM and DK (*F*_2,27_ = 14.1, *p* << 0. 001; Figure 1A, F). Nighttime emergence was significantly different in the three species and DA showed highest nocturnal emergence (LD-F_2,27_ = 74.4, *p* << 0. 001; Figure 1G). We also estimated the intra-species measure of day-to-day variation in phase of emergence peak as a read-out of the accuracy of the emergence peak and did not detect any difference in this measure among the three species (Figure 1H). The assay was conducted under LD twice with similar outcome (data not shown). Thus under LAB conditions the emergence rhythm of these three species differed from each another in terms of the onset of emergence, peak of emergence, nighttime emergence and gate-width, although many other features of the rhythm were similar across species.

### Seasonal variations in temperature and humidity

**Figure 2:**
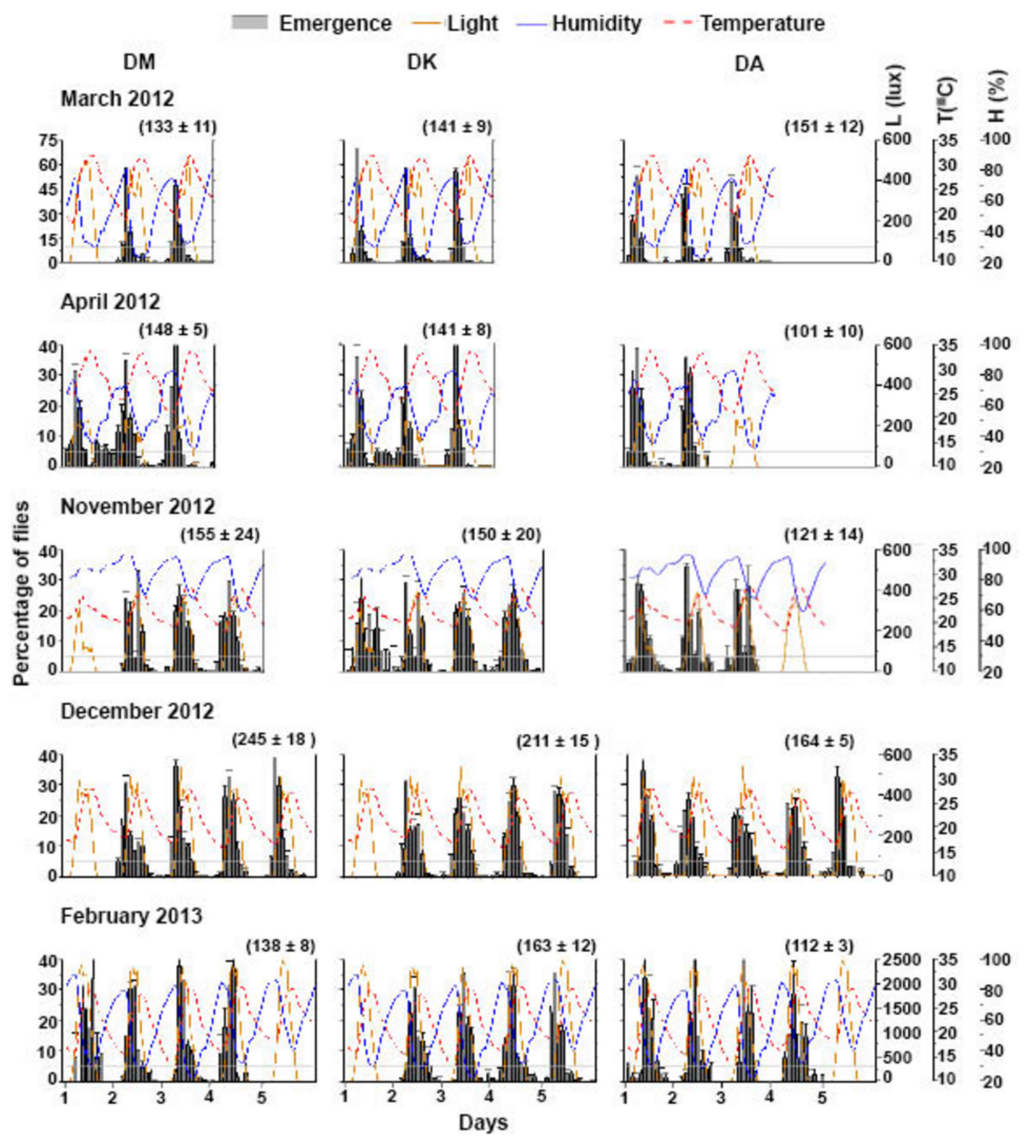
Seasonal variation in adult emergence rhythm of three Drosophilids under semi-natural conditions (SN). Average profiles of adult emergence rhythm (percentage of flies emerged/ 2-hr ± SEM, for each species averaged across 10 vials) of DM, DK and DA during five different months of the years 2012 (March, April, November and December) and 2013 (February) under SN is plotted along with environmental factors light (orange-solid curve), temperature (red-dashed curve) and humidity (blue-solid curve). Values in parentheses indicate the total number of flies emerged averaged across 10 vials (± SEM).

Since the natural environment contains a large number of simultaneously varying time cues, we asked whether the emergence rhythm of these three species may adopt different phase-relationships with such cues thus exhibiting temporal separation between the species. We assayed the rhythm under SN during five different months between 2012 and 2013 representing summer and winter conditions at this latitude. During this study the weather conditions varied especially in terms of temperature and humidity although light intensity at the study site was not different in four out of the five assays. The extreme high intensity light in one of the assays was due to the clearing of canopy above the enclosure and does not reflect a season-specific change. The temperature and humidity conditions in the five months during which our study was performed are summarized in Tables 1and 2. March and April-2012 were the warmest with maximum temperatures above 30 °C, and average daytime temperatures between 25 and 30 °C. These months also had lowest humidity levels. In November, humidity remained high throughout with average day and nighttime humidity above 80%. On the contrary, the average day and nighttime humidity in the other three months were around 60%. Due to a technical fault, humidity was not recorded in the month of December-2012. As expected the amplitude of daily oscillation in temperature was low in winter compared to summer, whereas phase of light onset and humidity trough remained mostly unaffected by season (Table 1, Figure 2). Since light intensity varied greatly depending on the extent of canopy it was not used to assess how harsh or mild the weather was in a particular month. Based on the temperature and humidity values, March and April conditions were considered as harsh and November, December and February as mild.

**Table 1:** Maximum and minimum of environmental factors across days (mean ± SEM).

	**Light intensity (lux)**	**Temperature (°C)**		**Humidity (%)**	
**Assay**	**Max**	**Max**	**Min**	**Max**	**Min**
**March 2012**	482.0 ± 16.5	31.7 ± 0.1	18.7 ± 0.6	79.3 ± 2.2	27.3 ± 2.7
**April 2012**	237.7 ± 5.8	33.5 ± 0.2	22.2 ± 0.9	79.7 ± 2.0	34.3 ± 1.8
**November 2012**	359.0 ± 16.9	25.1 ± 0.8	20.2 ± 0.2	94.3 ± 1.1	69.5 ± 4.5
**December 2012**	484.2 ± 17.6	27.2 ± 0.2	16.6 ± 0.3	-	-
**February 2013**	2375.0 ± 28.5	28.7 ± 0.3	15.7 ± 0.9	84.4 ± 1.9	30.0 ± 2.9

Humidity values for December 2012 were not collected due to a technical fault.

**Table 2:** Average values of environmental factors during day and nighttime across days (mean ± SEM). -Humidity values for December 2012 were not collected due to a technical fault.

	**Light intensity (lux)**	**Temperature (°C)**		**Humidity (%)**	
**Assay**	**Average day**	**Average day**	**Average night**	**Average day**	**Average night**
**March 2012**	257.4 ± 16.8	27.0 ± 0.1	23.1 ± 0.2	42.4 ± 2.6	61.8 ± 5.5
**April 2012**	142.2 ± 4.8	29.6 ± 0.3	25.5 ± 0.2	51.3 ± 0.9	63.9 ± 1.6
**November 2012**	171.7 ± 18.2	21.8 ± 0.4	22.3 ± 0.6	81.2 ± 2.4	87.3 ± 0.6
**December 2012**	239.2 ± 3.7	23.1 ± 0.3	19.6 ± 0.1	-	-
**February 2013**	1138.8 ± 23.5	24.0 ± 0.3	19.9 ± 0.7	49.9 ± 3.2	61.8 ± 3.3

### The three species responded similarly to seasonal variations in the natural environment

**Figure 3:**
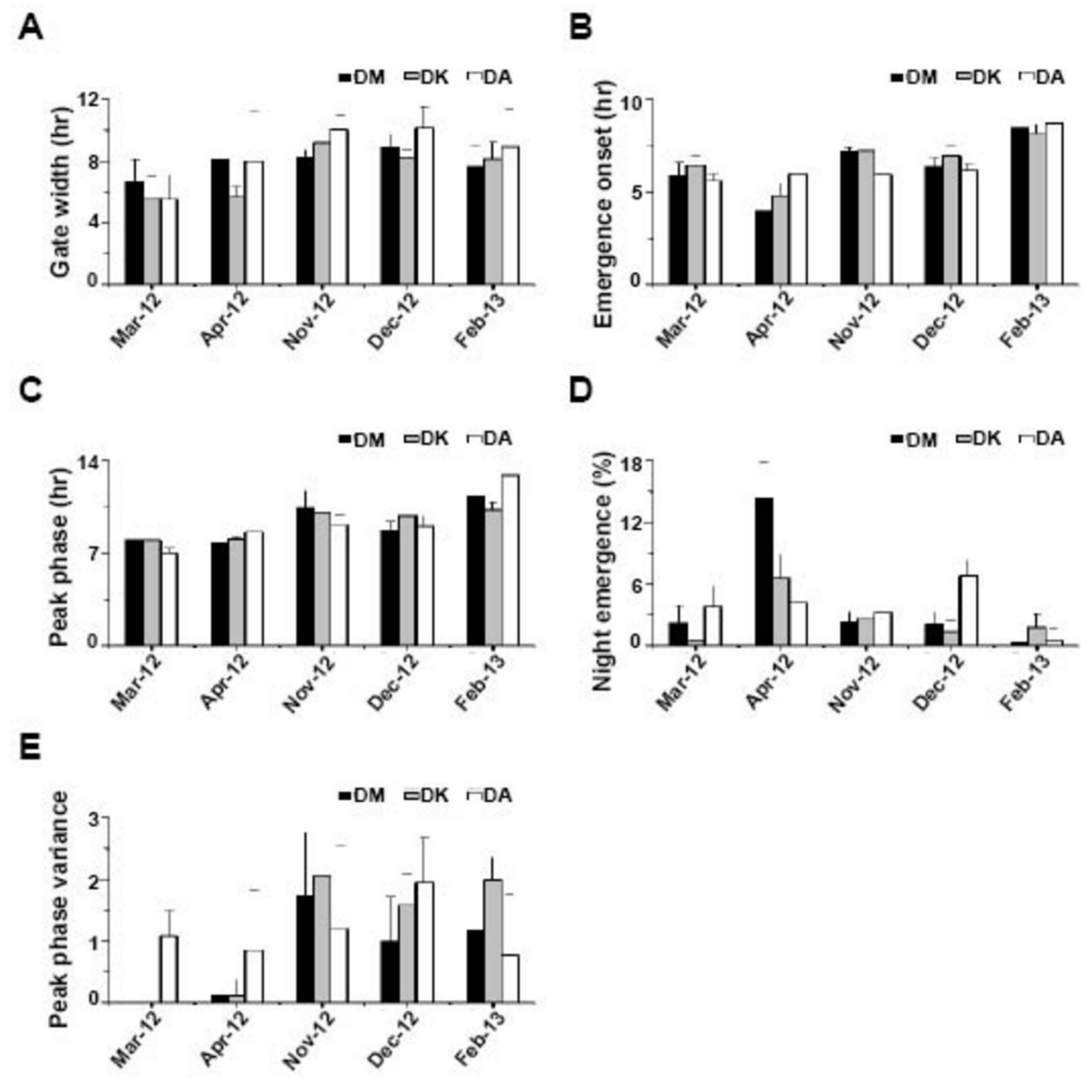
Properties of adult emergence rhythm of three Drosophilid species under semi-natural conditions (SN). (A) Average gate-width of emergence (averaged across 10 vials) of DM, DK and DA. (B) Average phase of onset of adult emergence (external time, averaged across 10 vials) of DM, DK and DA. (C) Average phase of adult emergence peak (external time, averaged across 10 vials) of DM, DK and DA. (D) Average percentage of nighttime emergence of flies (averaged across 10 vials) of DM, DK and DA. (E) Day-to-day variance in peak phase of emergence (averaged across 10 vials) of DM, DK and DA. Error bars are 95% Confidence Interval (± 95% CI).

We measured several properties of emergence rhythm in the three species across five different months under SN (Figures 2, 3). DM, DK and DA exhibited similar emergence pattern under SN except that during certain months all the three species showed some variations with respect to the number of cycles of emergence (Figure 2). Furthermore, a delay in initiation of emergence was seen in some months for DM (March, November, December) and DK (December, February). We did not see any consistent pattern in this delay based on the three environmental factors that we monitored. Unlike LAB assays, gate-width of emergence was not different among species but under SN, differed across months (narrower under harsh seasons; *F*_4,124_ = 15.5, *p* << 0. 001; Figures 2, 3A). We found that increase in temperature is associated with narrower gate-width of emergence in DK and DA- gate-width in DK and DA showed negative correlation with T_max_ (DK- *r* = −0.7; DA- *r* = −0.5, *p* < 0.05). High humidity levels probably enabled DK and DA flies to emerge in a broader window during the day- gate-width in DK and DA showed positive correlation with H_max_ (DK- *r* = 0.6; DA- *r* = 0.6, *p* < 0.05). Although humidity is usually inversely correlated with temperature we cannot rule out the combined action of the two. For DM, such correlations of temperature and humidity with gate-width did not reach statistical level of significance. Onset of emergence was clearly affected by season (*F*_4,124_ = 69.7, *p* << 0. 001), as evidenced by the delayed onset in February-2013 for all the three species with no difference between them (Figures 2, 3B). As temperature increased, there was an advance in the phase of onset of emergence in all the three species- onset of emergence showed a negative correlation with T_min_ and T_ave night_ (T_min_ DM- *r* = −0.6, DK- *r* = −0.6; DA- *r* = −0.7; T_ave night_ DM- *r* = −0.6; DK- *r* = −0.5; DA- *r* = −0.7, *p* < 0.05). Similar to onset of emergence, peak of emergence was also affected by season (F_4,124_ = 73.9.7, *p* << 0.001) and there was no difference among the three species (Figures 2, 3C). Peak of emergence was also advanced with increase in nighttime temperature- peak of emergence showed negative correlation with T_min_ and T_ave night_ (T_min_ DM- *r* = −0.7; DK- *r* = −0.8; DA- *r* = −0.8; T_ave night_ DM- *r* = −0.8; DK- *r* = −0.7; DA- *r* = −0.7, *p* < 0.05). We found that increased nighttime humidity was associated with an advance in the phase of emergence-peak (H_ave night_ DM- *r* = −0.7; DK- *r* = −0.7; DA- *r* = −0.7, *p* < 0.05) although we cannot conclude a causal role for humidity levels in modulating emergence from these results. During November, December-2012 and February-2013, the peak of emergence shifted towards the day probably because favourable conditions persisted past dawn (Figures 2, 3C). Our studies show that nighttime emergence was greater across species during comparatively warmer and drier days (except in DA during December-2012) (Figures 2, 3C). There was no difference among species in the nighttime emergence which differed among months (*F*_2,129_ = 36.6, *p* << 0. 001; Figure 3C). Fraction of flies emerging in the nighttime was significantly higher in the month of April-2012 compared to all other months (including March-2012) in case of DM and DK, while DA had similar fraction of flies emerging during nighttime across months (Figure 3C).

Day-to-day variation in peak timing was greater in relatively milder conditions especially for DM and DK (Figures 2, 3E) and does not differ among species in any given month, although it did differ across months (*F*_4,116_ = 16.16, *p* << 0. 001). A significant interaction between species and months (*F*_8,125_ = 3.64, *p* < 0.001) was detected probably due to the fact that unlike DM and DK flies, DA did not show any reduction in variance in the harsher months of March and April (Figures 2, 3E). Since the reduction in day-to-day variance in phase of peak emergence could be a trivial consequence of reduced variation in environmental conditions, we examined variance in the T_ave day_ during all the five months and found that there was no such reduction during the harsh months (similar SEM values, Table 2). As the weather conditions became milder between November-2012 to February-2013, the amplitude of peak of emergence was reduced (*F*_4,129_ = 74.97, *p* < 0.001). Amplitude of the peak also differed across species (*F*_2,129_ = 4.38, *p* < 0.01) with significant interaction between species and months (*F*_8,129_ = 6.92, *p* < 0.001). This reduction in the peak amplitude can be considered as a by-product of broadening of the gate-width of emergence under milder conditions. 

In summary, adult emergence rhythm of the three species (DM, DK and DA) differ under LAB (LD and DD), however, such differences were not detectable in SN. This is possibly due to the presence of stronger and richer time cues in nature. 

## Discussion

Although early studies on circadian rhythms in insects employed a wide variety of species [2], over the past few decades *D. melanogaster* has become the most widely used Drosophilid to study circadian clocks due to the development of various genetic tools and the availability of mutants. More recently few studies have explored other Drosophilids which shed some light on how they differ among each other in terms of their clock properties and rhythmic behaviour [3,4,14,15]. We investigated adult emergence behaviour in three closely related Drosophilids [16,17], under various environmental conditions in both LAB (LD and DD) and SN (harsh and mild seasons) to investigate whether there is any inter-species difference in their emergence patterns. Although differences in mating or feeding are more likely to promote speciation we reasoned that the differences in activity may be a reflection of the ability of DA flies to tolerate harsh environmental conditions of mid day and that therefore DA may have also differed in their emergence pattern. Our studies were carried out on three species of Drosophilids that have been relatively recently (2004-2005) caught from the wild, from locations within Bangalore, India and therefore can be considered sympatric; however, the possibility that they occupy different micro-habitats cannot be ruled out. Previously we have reported the temporal separation of activity rhythm in DM and DA under LAB and SN [3,4], here we report that the adult emergence rhythm differs only in the LAB. Furthermore, while we had reported earlier that free-running period of activity-rest rhythm of DM is greater than DA [3], this difference did not extend to the period of adult emergence rhythm (Figure 1D). Moreover, DM and DK showed almost similar adult emergence pattern both under LD and DD (Figure 1) much like their activity/rest pattern [4]. Even though there are no studies to the best of our knowledge that unravel the phylogenetic relationship between DM and DK, it is clear from our studies that these two species may have similar circadian organization.

When studied under SN at five different months, the interspecies differences in the adult emergence rhythm reduced to a great extent. However, they all showed changes in their emergence rhythm consistent with variations in environmental conditions and they responded to changes in the environment very similarly. This is not surprising in the light of recent studies on activity-rest rhythm in which factors in natural environment was shown to dominate the behaviour more than the genotype and even the circadian clock mutant flies *per* ^0^ showed activity-rest pattern very similar to the wild type flies [9-11]. In case of adult emergence also, the arrhythmicity in *per* ^0^ mutants seen in the LAB was partly rescued under SN [6]. Here we show that even if such differences in emergence rhythm exist among the three related species of *Drosophila*, they are overridden by natural environmental factors. However, in another long-term study in which we assayed the activity/ rest rhythm of these three species under SN across seasons over a span of 1.5 yrs revealed that DA continued to be diurnal similar to their LAB behaviour, suggesting that the overwhelming effect of natural environment cannot not be generalised to all circadian behaviours [4].

Temperature appears to play a major role in gating adult emergence rhythm in *Drosophila*, and under harsh or high temperature-low humidity conditions, flies of all three species avoid emerging during the later part of the day similar to previous reports on DM [6]. We find that gate-width of only DK and DA was reduced with increasing temperature while this was not apparent in DM. It is likely that this reduction is due to the high amplitude cycling of temperature during the warmer months of March and April in contrast to November. While a previous LAB study on DM has shown that low amplitude warm/cold cycle (29/25 °C) does not alter the gate-width from that of a constant 25 °C regime [12], higher amplitude cycles (28/18°C) can reduce gate-width by ~4 hours (Nikhil KL and Sharma VK, personal communication). Yet another study has shown both theoretically and empirically that gate-width of DM is likely to widen with increase in ambient temperature [18]. Our studies reveal that under natural conditions, across months where temperature fluctuated as high as13 °C (March, February) or as low as 5 °C (November), gate-width was not significantly altered. Onset and peak of emergence was also affected by temperature and humidity in such a way that during drier and hotter days, flies emerged earlier perhaps to avoid harsh conditions [6]. Even though humidity levels showed significant correlation with the emergence properties, we acknowledge that its values recorded from the enclosure may not reflect those inside the glass vials in which the pupae developed, due to the constant presence of food medium. Unlike previous studies under SN, our study did not show correlation of light with any of the features of the emergence rhythm [6]. This could be because in our assays, light intensity did not vary much across the months (except February-2013, Tables 1, 2).

Thus our studies performed under both LAB and SN on the adult emergence rhythm of three closely related Drosophilids - DM, DK and DA, suggests that (1) inter-species differences in the properties of one circadian behaviour need not be reflected in another, (2) the difference in a particular rhythmic behaviour seen under the simplified LAB environment may not manifest under SN due to overriding effects of strong natural time cues. This also underscores the point that while studying behaviour of species under more natural-like conditions one must exercise caution in interpreting the results as it is not easy to separate the clock-controlled phenotypes from mere masking due to the presence of multiple strong environmental factors. 

## Supporting Information

Figure S1 **DK flies show activity/ rest pattern similar to DM.** (**A**) Raw Activity counts (15 min bin) averaged across 5 days for DM (black-solid curve), DK (red-dotted curve) and DA (blue-solid curve) virgin male flies (mean ± SEM). (**B**) Average actograms of DK virgin male flies under 6 days of LD12:12 followed by DD. Grey shaded areas indicate darkness.
(TIF)
