# Supplementary figures and images for: Natural Conditions Override Differences in Emergence Rhythm among Closely Related Drosophilids
Source: PLoS One. 2013 Dec 11;8(12):e83048. doi: 10.1371/journal.pone.0083048 (PMC3859640; doi:10.1371/journal.pone.0083048)

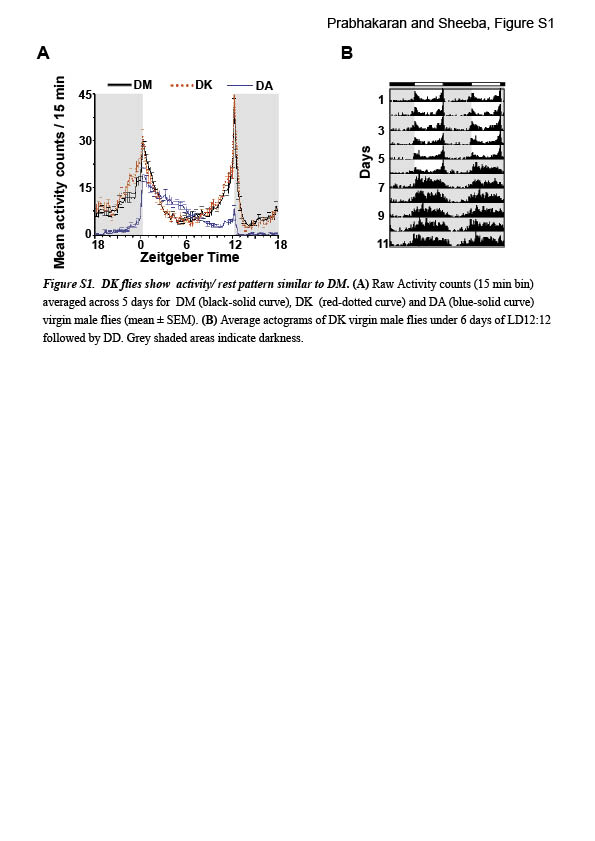

Supplement: Figure S1 — DK flies show activity/ rest pattern similar to DM. (A) Raw Activity counts (15 min bin) averaged across 5 days for DM (black-solid curve), DK (red-dotted curve) and DA (blue-solid curve) virgin male flies (mean ± SEM). (B) Average actograms of DK virgin male flies under 6 days of LD12:12 followed by DD. Grey shaded areas indicate darkness. (TIF) [file pone.0083048.s001.tif]
